# Polymer-mediated surface engineering enables directional growth of RuO_2_ nanorods on electrospun V_2_O_5_ nanofibers

**DOI:** 10.3762/bjnano.17.106

**Published:** 2026-11-04

**Authors:** Jiwoo Song, Namhee Kim, Minjae Kim, Myung Hwa Kim, Dasol Jin

**Affiliations:** 1 Department of Chemistry & Nanoscience, Ewha Womans University, Seoul 03760, Republic of Korea; 2 Institute of Multiscale Matter and System (IMMS), Ewha Womans University, Seoul 03760, Republic of Korea; 3 Department of Chemistry, Jeonbuk National University, Jeonju 54896, Republic of Korea

**Keywords:** anisotropic crystal growth, electrospinning, polymer engineering, RuO_2_ nanorods, V_2_O_5_ nanofibers

## Abstract

Controlling the morphology of metal oxide nanostructures through simple and scalable synthetic strategies remains challenging because crystal growth is strongly governed by interfacial surface chemistry. Herein, we present a polymer-mediated strategy for directing the growth of RuO_2_ nanorods on electrospun V_2_O_5_ nanofibers by tuning the polyvinylpyrrolidone (PVP)/polyacrylonitrile (PAN) ratio in the precursor solution. PAN incorporation preserves the one-dimensional V_2_O_5_ framework while progressively promoting the formation of vertically aligned RuO_2_ nanorods. Structural and spectroscopic analyses reveal that PAN-induced thermal evolution modifies the surface chemistry of V_2_O_5_ by increasing pyridinic nitrogen functionalities and oxygen-deficient surface sites. These surface characteristics are proposed to facilitate preferential Ru precursor nucleation, leading to anisotropic crystal growth without altering the crystal structures of either V_2_O_5_ or RuO_2_. The optimized composition containing 50 wt % PAN exhibits the most homogeneous and vertically aligned RuO_2_ nanorod arrays.

## Introduction

Controlling crystal growth at heterogeneous oxide interfaces remains a fundamental challenge because nucleation and morphological evolution are highly sensitive to the local chemical environment during thermal processing [1]. Although numerous synthetic approaches have been developed to regulate metal oxide nanostructures, precise control over anisotropic crystal growth generally relies on surfactants, templates, or complex solution chemistries that limit scalability and synthetic simplicity [2]. Vanadium oxides (VO*_x_*, *x* = 1.0–2.5) exhibit multiple accessible oxidation states (V^3+^, V^4+^, and V^5+^), enabling reversible redox reactions for a wide range of electrochemical energy-storage and conversion applications [3–7]. Despite these advantages, their relatively poor electrical conductivity often limits reaction kinetics and overall electrochemical performance. Hybridization with highly conductive noble metal oxides provides an attractive strategy to overcome these limitations by simultaneously improving charge transport and interfacial electron transfer. Among various candidates, RuO_2_ is particularly attractive owing to its metallic conductivity, excellent chemical stability and fast surface redox kinetics, making it one of the most efficient oxide materials for electrocatalysis [8–9], electrochemical sensing [10–12], and heterogeneous catalytic processes [13–14].

Existing synthetic approaches predominantly rely on surfactants, templates, or ligands to manipulate crystal growth [15–16]. While these methods effectively control morphology, they often increase synthetic complexity and provide limited control over the coupled evolution of oxide supports and secondary crystal nucleation during thermal processing. Consequently, developing a simple and scalable strategy that regulates crystal growth through intrinsic interfacial chemistry, rather than external structure-directing agents, remains highly desirable. In this regard, polymer-assisted electrospinning provides a unique opportunity to regulate interfacial chemistry during oxide formation [17–18]. Beyond controlling the morphology of precursor fibers, polymer composition governs the thermal evolution of the precursor matrix, thereby determining the formation of residual surface functionalities and defect structures after calcination. These polymer-derived surface characteristics can significantly influence heterogeneous nucleation and subsequent crystal growth; however, their role in directing the morphology of secondary metal oxides remains largely unexplored [19].

Herein, we demonstrate that polymer composition can be exploited to direct the anisotropic growth of RuO_2_ nanorods on electrospun V_2_O_5_ nanofibers. By systematically tuning the polyvinylpyrrolidone (PVP)/polyacrylonitrile (PAN) ratio, the thermal evolution of the precursor fibers is regulated while preserving the one-dimensional V_2_O_5_ framework after calcination. The resulting polymer-derived surface chemistry, characterized by increased pyridinic nitrogen functionalities and oxygen-deficient surface sites, is proposed to facilitate preferential Ru precursor nucleation and anisotropic crystal growth, leading to uniformly aligned nanorods without altering the crystal structures of either V_2_O_5_ or RuO_2_.

## Experimental

### Materials

Vanadium(III) chloride (VCl_3_, 97%), ruthenium(III) chloride hydrate (RuCl_3_·*x*H_2_O), polyvinylpyrrolidone (PVP, *M*_W_ ≈ 1 300 000) and polyacrylonitrile (PAN, *M*_W_ ≈ 150 000) were purchased from Sigma-Aldrich (St. Louis, MO, USA). *N*,*N*-dimethylformamide (DMF, extra pure grade), ethanol (94.5%, extra pure grade), and sodium hydroxide (NaOH) beads were purchased from Ducksan (Korea). All other chemicals used were of analytical grade without further purification, and all solutions used for experiments were prepared with deionized water (resistivity ≥ 18 MΩ·cm).

### Synthesis

Vanadium oxide nanofibers were synthesized via electrospinning followed by post-annealing, and the resulting samples were denoted as VF(*x*), where *x* represents the PAN weight percentage (wt %) in the precursor polymer composition. For VF(0), total of 50 mg of metal precursors were dissolved in a 1:1 (v/v) mixture of ethanol and deionized water. The resulting solution was ultrasonicated for 10 min, and then 300 mg of PVP was added. The mixture was stirred at 25 °C for 24 h to ensure complete homogenization and viscosity optimization for electrospinning. The as-prepared solution was then loaded into 12 mL plastic syringe fitted with a 25-gauge needle. Electrospinning (NanoNC ESR200R2) was performed under the following conditions: flow rate of 5 μL·min^−1^, applied voltage of 15.0 kV, and needle-to-collector distance of 11 cm. VF(30), VF(50), VF(70), and VF(100) were prepared by incorporating PAN at 30, 50, 70, and 100 wt %, respectively. All the resulting as-spun nanofibers were calcined at 350 °C under a gas flow of 20 sccm O_2_ and 80 sccm He, with ramping rate of 0.5 °C·min^−1^. After synthesizing the VF(*x*) samples, amorphous ruthenium hydroxide (i.e., Ru(OH)*_y_*) was prepared by the dropwise addition of 0.1 M NaOH to an aqueous RuCl_3_·*x*H_2_O solution until the pH reached 10 at 25 °C. The resulting precipitate was thoroughly washed with deionized water and redispersed in ethanol to form a homogeneous suspension. The Ru(OH)*_y_* suspension was then drop-cast onto the VF(*x*) substrates, followed by drying for 30 min. Subsequently, the samples were annealed in air at 200 °C for 3 h to obtain RuO_2_. The resulting composites were denoted as RuO_2_–VF(*x*).

### Physical characterization

Morphological and elemental analyses were conducted via field-emission scanning electron microscopy (FESEM, JEOL JSM-7610F). High-resolution structural features were examined using high-resolution transmission electron microscopy (HRTEM, FEI Tecnai F20) and transmission electron microscopy (TEM, JEOL JEM-ARM200F NEOARM). Surface chemical states were investigated by angle-resolved X-ray photoelectron spectroscopy (ARXPS, Thermo Fisher Scientific K-ALPHA XPS, Al Kα radiation at 12 kV) and vibrational properties were probed using Raman microscope (HORIBA LabRAM HR Evolution 800). Thermal analysis was conducted with simultaneous thermogravimetric and differential thermal analysis (TGA-DSC, TA instruments STD 650) with a heating rate of 10 °C·min^−1^ up to 800 °C in air. Crystal structures were analyzed by X-ray diffraction (XRD; Malvern Panalytical) using Cu Kα radiation (λ = 1.5406 Å).

## Results and Discussion

**Figure 1:**
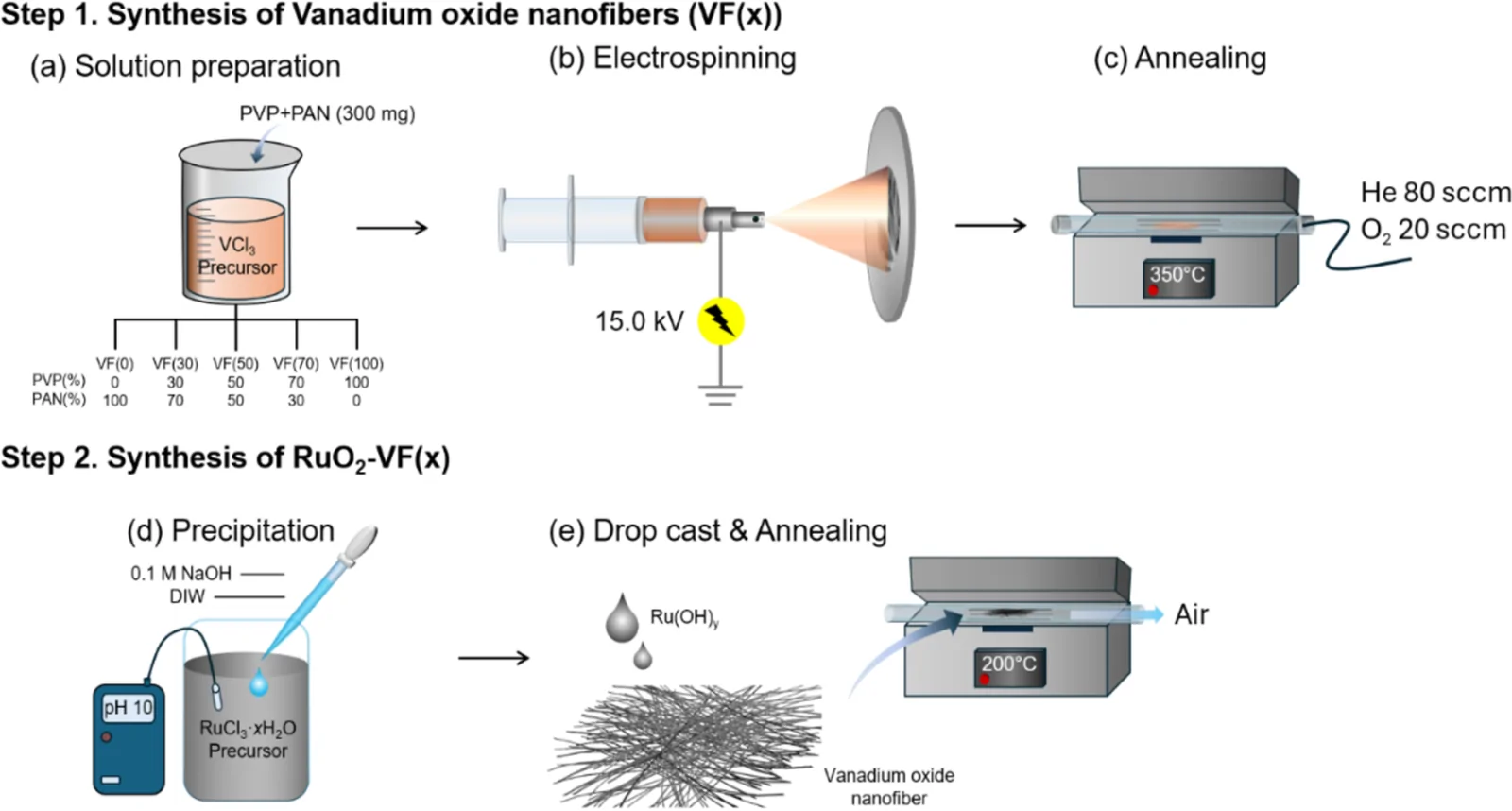
Schematic illustration of the synthetic methodology for RuO_2_–VF(x) nanostructures.

**Figure 2:**
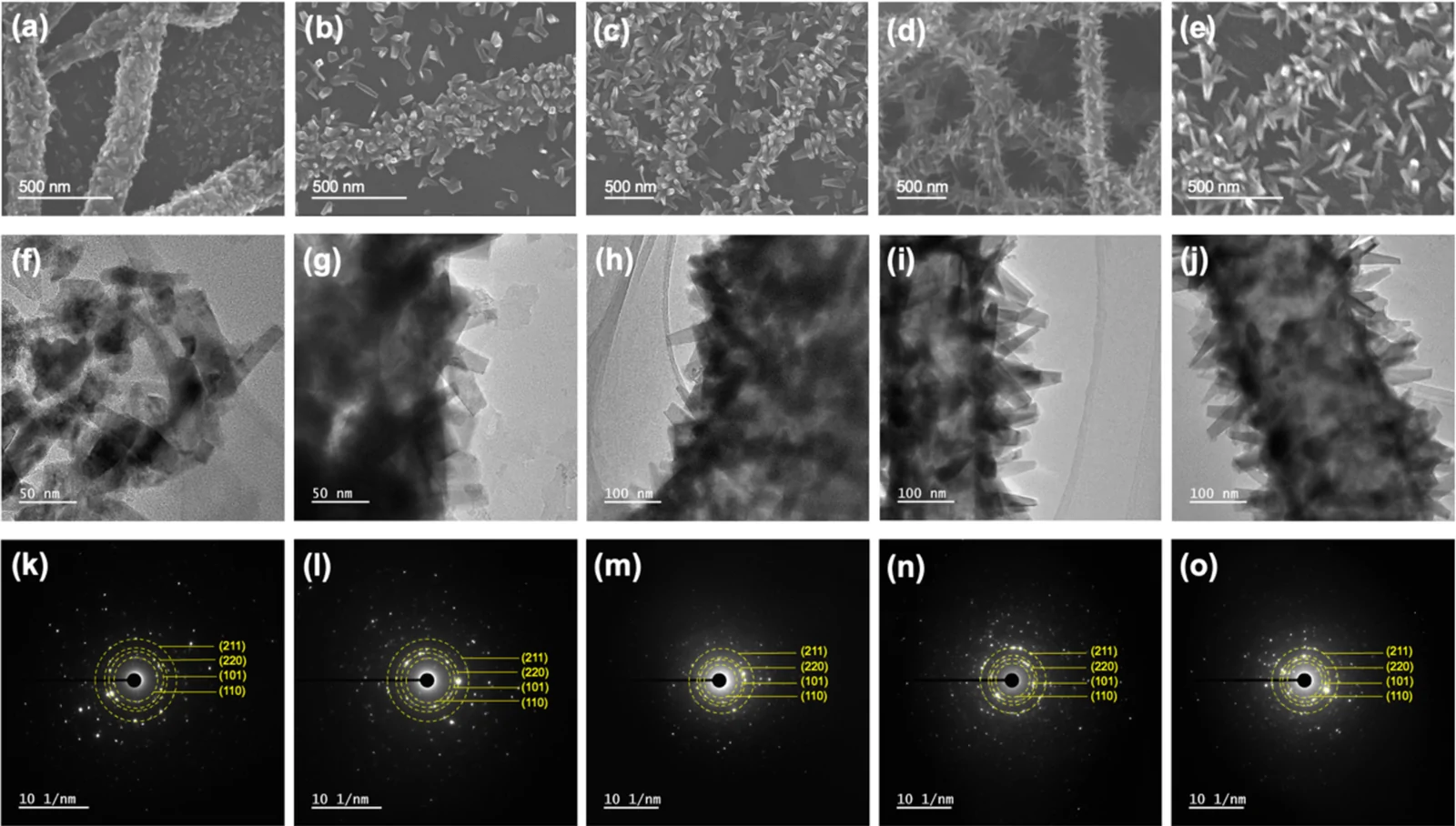
Morphological evolution of RuO_2_ nanorods on electrospun V_2_O_5_ nanofibers with increasing PAN content (from left to right: *x* = 0, 30, 50, 70, and 100 wt %): (a–e) SEM images, (f–j) corresponding TEM images, and (k–o) SAED patterns of RuO_2_–VF(*x*). The diffraction rings are indexed to the (110), (101), (220), and (211) planes of rutile RuO_2_ (JCPDS No. 65-2824).

To elucidate the influence of polymer composition on the growth behavior of RuO_2_, the morphology and microstructure of the RuO_2_–VF(*x*) composites were systematically characterized. The corresponding fabrication scheme is shown in Figure 1. SEM images (Figure 2a–e) reveal that the electrospun V_2_O_5_ nanofiber framework is well preserved over the entire PAN composition range, indicating that the incorporation of PAN effectively suppresses structural collapse during thermal processing. In the absence of PAN (RuO_2_–VF(0), Figure 2a), RuO_2_ is randomly deposited as discontinuous particulate aggregates on the nanofiber surface, suggesting that crystal nucleation occurs isotropically without a preferred growth direction [20–21]. Upon increasing the PAN content, however, the RuO_2_ growth mode gradually transitions from randomly distributed nanoparticles to vertically aligned nanorods: At 30 wt % PAN, elongated RuO_2_ nanorods become discernible, while increasing the PAN content to 50 wt % results in densely packed and uniformly aligned nanorod arrays covering the V_2_O_5_ nanofiber surface. Further increasing the PAN content to 70 and 100 wt % produces no significant changes in nanorod morphology, indicating that the conditions favoring anisotropic RuO_2_ growth are achieved at approximately 50 wt % PAN.

TEM observations (Figure 2f–j) further corroborate the SEM results by revealing the microstructural evolution of the RuO_2_ coating. While RuO_2_–VF(0) exhibits a discontinuous layer of randomly distributed nanoparticles, PAN incorporation promotes the formation of radially oriented RuO_2_ nanorods with progressively improved uniformity and alignment. The RuO_2_–VF(50) sample displays the most homogeneous nanorod architecture, supporting the conclusion that polymer composition governs the transition from isotropic particle deposition to anisotropic crystal growth. The crystal structure of the deposited RuO_2_ was further examined by selected-area electron diffraction (SAED) (Figure 2k–o). All samples exhibit well-defined diffraction rings indexed to the (110), (101), (220), and (211) planes of rutile RuO_2_ (JCPDS No. 65-2824), confirming the formation of crystalline RuO_2_ regardless of PAN content. Despite the significant morphological evolution observed by SEM and TEM, the diffraction patterns remain essentially unchanged, indicating that polymer modulation regulates the nucleation and growth behavior of RuO_2_ without altering its intrinsic crystal structure. To further investigate the elemental distribution within the RuO_2_–V_2_O_5_ nanostructures, TEM-EDS elemental mapping was performed on RuO_2_–VF(0) and RuO_2_–VF(50) as representative samples (Supporting Information File 1, Figure S1 and Figure S2). Consistent with the SEM observations, RuO_2_–VF(0) exhibits a rough surface, whereas RuO_2_–VF(50) features distinct RuO_2_ nanorods grown on V_2_O_5_ nanofibers. The detection of C, Ru, N, and V in both samples further supports the formation of RuO_2_/V_2_O_5_ heterostructures. The XRD patterns in Figure S3 (Supporting Information File 1) show characteristic reflections of rutile RuO_2_, indexed to the (110), (101), (220), and (211) planes (JCPDS No. 65-2824), consistent with the TEM-SAED results. The VF(100) PVP-100 sample exhibits relatively lower peak intensity than the PAN/PVP mixed and PAN-only samples.

**Figure 3:**
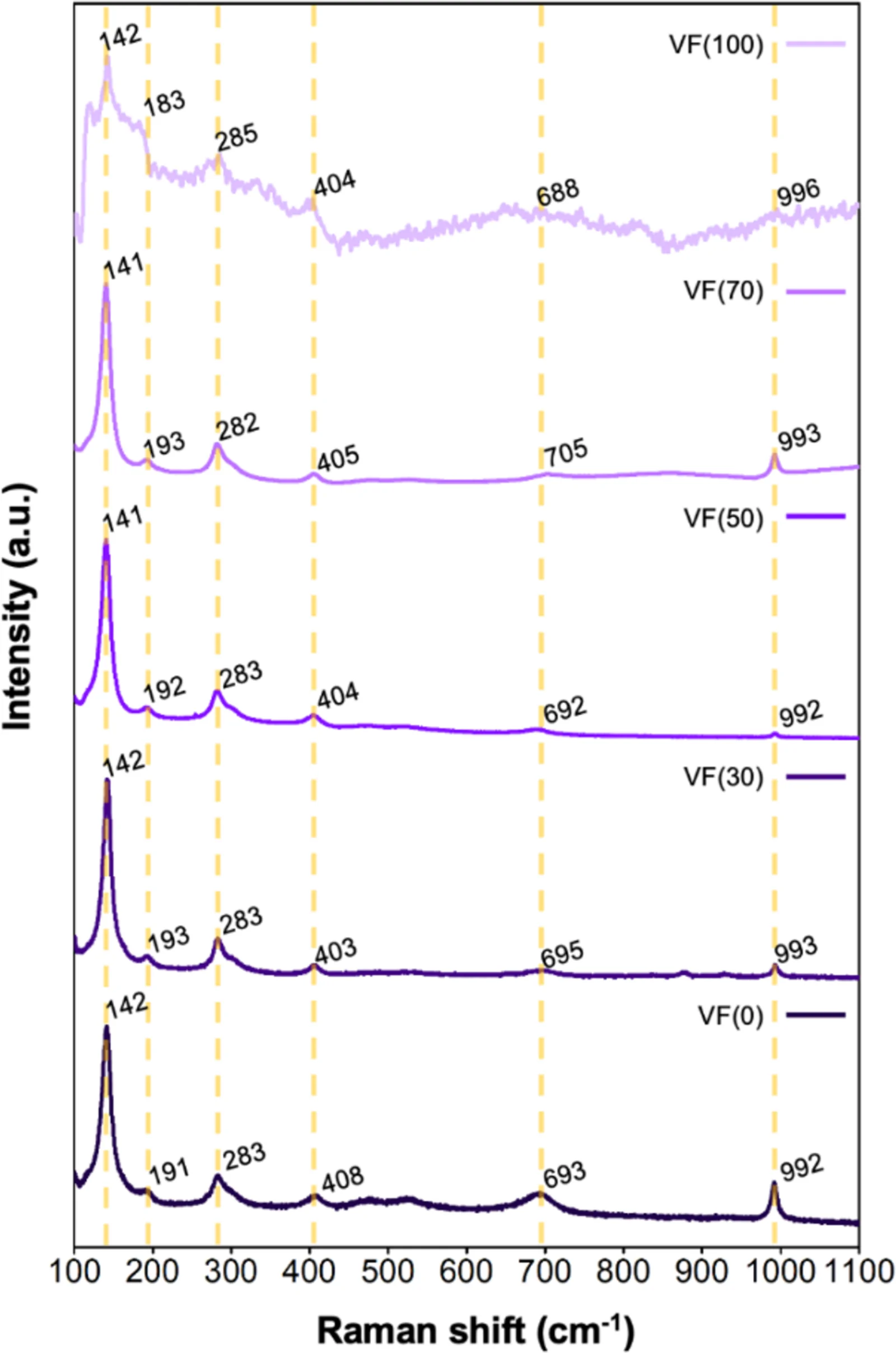
Raman spectra of electrospun V_2_O_5_ nanofibers (VF(*x*)) with increasing PAN content (*x* = 0, 30, 50, 70 and 100 wt %), demonstrating the preservation of the orthorhombic α-V_2_O_5_ structure.

To elucidate the structural characteristics of the V_2_O_5_ substrate governing the subsequent growth of RuO_2_ nanorods, Raman spectroscopy was performed on the synthesized VF(*x*) nanofibers (Figure 3). All samples exhibit the characteristic Raman modes of orthorhombic α-V_2_O_5_, confirming the successful formation of the crystalline V_2_O_5_ framework regardless of polymer composition. The characteristic bands at approximately 142, 192, 284, 405, 698, and 994 cm^−1^ are assigned to the B_1_g/B_3_g lattice vibration, Ag/B_2_g vanadium vibration, V–O bending, Ag V–O rocking, B_2_g V–O stretching, and terminal V=O stretching modes, respectively, in good agreement with previous reports [22–25]. Notably, the positions of these characteristic Raman bands remain essentially unchanged from VF(0) to VF(70), indicating that increasing the PAN content does not alter the crystal structure of the electrospun V_2_O_5_ nanofibers. Although VF(100) exhibits reduced peak intensity accompanied by an increased spectral background, the characteristic α-V_2_O_5_ Raman modes remain clearly identifiable, suggesting that the crystalline framework is largely preserved even at the highest PAN content. These results demonstrate that modulation of the PVP/PAN ratio primarily influences the surface characteristics of the nanofibers without inducing phase transformation of the V_2_O_5_ substrate.

**Figure 4:**
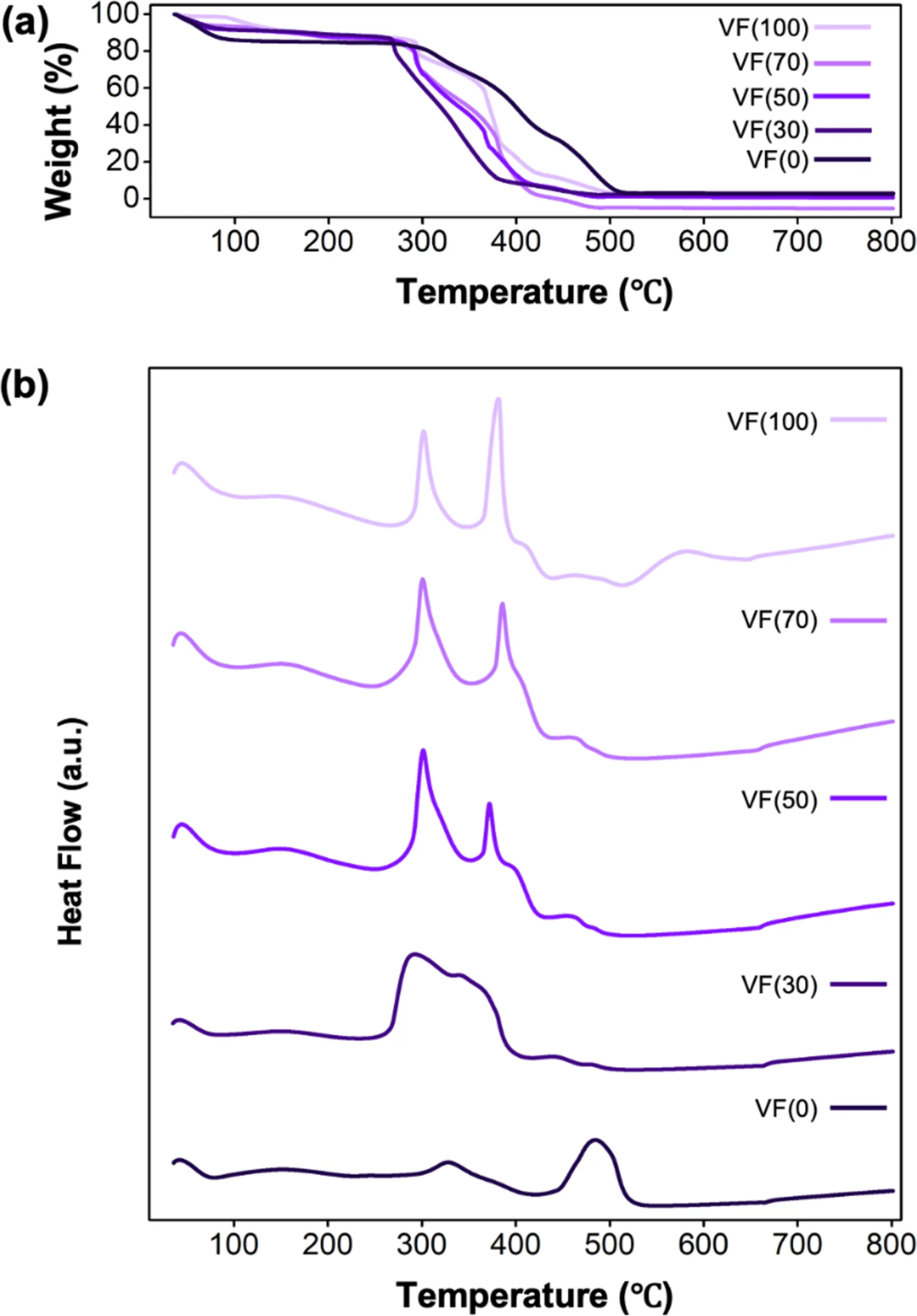
Thermal evolution of electrospun VF(*x*) nanofibers as a function of PAN content (*x* = 0, 30, 50, 70 and 100 wt %): (a) TGA curves and (b) DSC thermograms.

To understand how polymer composition influences the growth behavior of RuO_2_ nanorods, the thermal evolution of the as-spun VF(*x*) nanofibers was investigated by thermogravimetric analysis (TGA) and differential scanning calorimetry (DSC) in air (Figure 4). A notable feature observed in both TGA and DSC is the nearly identical thermal profiles of VF(*x*) with PAN contents of 50–100 wt %, which coincide with the saturation of the RuO_2_ morphology observed by SEM and TEM (vide supra). This correlation suggests that the thermal evolution of the polymer matrix plays a critical role in establishing the interfacial chemical environment that governs the nucleation and anisotropic growth of RuO_2_. As shown in Figure 4a, all samples exhibit an initial weight loss below 100 °C, which is attributed to the evaporation of residual solvent and physically adsorbed moisture. The thermal decomposition behavior thereafter strongly depends on polymer composition. VF(0), consisting solely of PVP, exhibits two major weight-loss events at approximately 330 and 445 °C, accompanied by corresponding exothermic peaks in the DSC profile (Figure 4b), which are associated with PVP side-chain decomposition and backbone combustion [26]. In contrast, PAN-containing samples exhibit distinctly different thermal characteristics. For VF(30), new exothermic events emerge at approximately 300 and 370 °C, corresponding to oxidative cyclization of nitrile groups and subsequent aromatization of the polymer backbone, respectively [27–28]. These reactions progressively dominate the thermal behavior as the PAN content increases, leading to nearly identical thermal profiles for VF(50–100). Importantly, the oxidative stabilization occurring near 300 °C takes place within the calcination temperature range employed for V_2_O_5_ nanofiber synthesis. The formation of a thermally stable ladder-like structure during this process is expected to suppress structural relaxation of the precursor fibers while simultaneously modifying the chemical environment of the oxide surface. The close agreement between the thermal evolution and the morphology of the resulting RuO_2_ suggests that PAN-derived thermal stabilization contributes to the formation of surface conditions favorable for anisotropic RuO_2_ growth.

**Figure 5:**
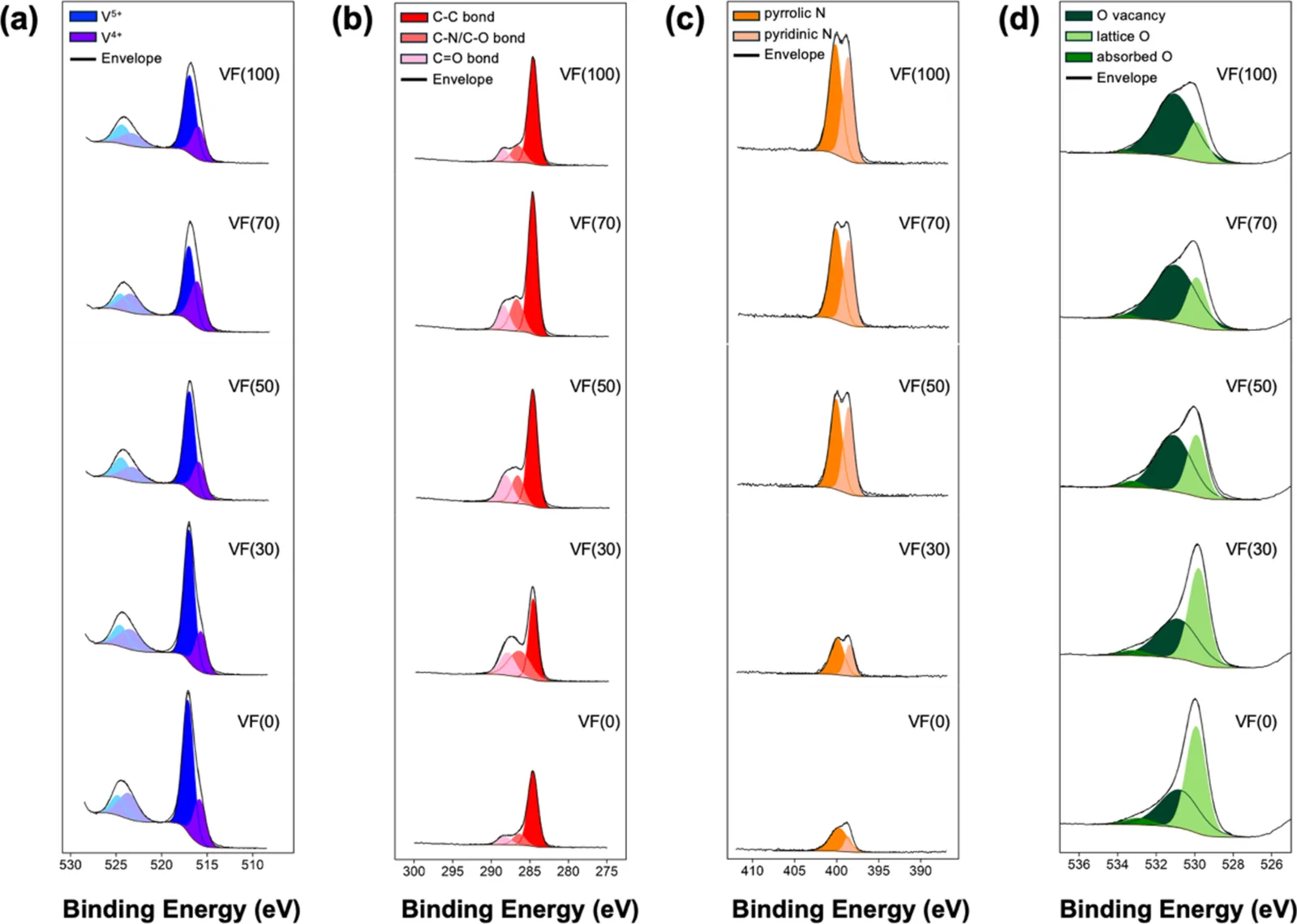
ARXPS spectra of VF(*x*) with increasing PAN content (*x* = 0, 30, 50, 70, and 100 wt %), showing the (a) V 2p, (b) C 1s, (c) N 1s, and (d) O 1s core-level regions.

To elucidate how polymer composition modifies the surface chemistry of the V_2_O_5_ support, X-ray photoelectron spectroscopy (XPS) was performed on the synthesized VF(*x*) nanofibers (Figure 5). The V 2p spectra (Figure 5a) exhibit characteristic V^4+^ and V^5+^ components at 515.9/523.2 and 517.0/524.3 eV, respectively, in good agreement with reported values for orthorhombic V_2_O_5_ [29–30]. No appreciable changes in either the binding energies or relative spectral features are observed with increasing PAN content, indicating that polymer modulation does not alter the chemical state of the V_2_O_5_ framework, consistent with the Raman results (vide supra). In contrast, the surface carbon and nitrogen chemistries exhibit pronounced changes with polymer composition. The C 1s spectra (Figure 5b) can be deconvoluted into C–C, C–N/C–O, and C=O components centered at 284.6, 286.5, and 288.0 eV, respectively [31–32]. More importantly, the N 1s spectra (Figure 5c) show that increasing the PAN content progressively enriches the V_2_O_5_ surface with pyridinic nitrogen functionalities, particularly above 50 wt %. These nitrogen functionalities originate from PAN-derived oxidative stabilization during calcination, in agreement with the thermal analysis. Since pyridinic nitrogen has been reported to serve as an effective anchoring site for Ru precursor species [33], the increased abundance of pyridinic nitrogen is expected to facilitate preferential Ru nucleation during the subsequent deposition process. The evolution of the O 1s spectra (Figure 5d) further supports the polymer-induced surface reconstruction. Deconvolution of the spectra identifies lattice oxygen, oxygen-deficient species, and adsorbed oxygen at approximately 530, 531, and 533 eV, respectively [34–35]. Notably, the relative concentration of oxygen-deficient surface species increases progressively with PAN incorporation, with a pronounced increase observed above 50 wt % PAN. Consistent with this trend, the oxygen-vacancy-to-lattice-oxygen ratio increases from approximately 1.2 for VF(0) and VF(30) to 1.8, 2.6, and 3.5 for VF(50), VF(70), and VF(100), respectively. The evolution of pyridinic nitrogen and oxygen-deficient surface sites closely parallels the morphological transition of RuO_2_ observed by SEM and TEM, suggesting that polymer-derived surface reconstruction modifies the interfacial chemical environment governing Ru precursor adsorption and anisotropic crystal growth. Furthermore, the V^4+^ species detected by XPS may facilitate the nucleation of rutile RuO_2_. Because V^4+^ can locally form rutile-type VO_2_-like domains, which are isostructural with RuO_2_ and have comparable lattice parameters, these domains could serve as low-mismatch templates for RuO_2_ nucleation [36].

## Conclusion

In this study, we developed a polymer-regulated strategy for simultaneously stabilizing electrospun V_2_O_5_ nanofibers and directing the anisotropic growth of RuO_2_ nanorods by tuning the PVP/PAN ratio in the electrospinning precursor. By systematically tuning the PVP/PAN ratio, the thermal evolution of the precursor fibers and the resulting surface chemistry were effectively controlled while preserving the crystalline V_2_O_5_ framework. In particular, a PAN content of approximately 50 wt % promotes the formation of pyridinic nitrogen functionalities and oxygen-deficient surface sites, which are proposed to facilitate preferential Ru precursor nucleation and the subsequent growth of uniformly aligned RuO_2_ nanorods. These findings demonstrate that polymer composition serves as an effective means of regulating interfacial chemistry during thermal processing, providing a simple and scalable strategy for controlling oxide nanostructures without relying on surfactants, templates, or complex synthetic procedures.

## Supporting Information

File 1 Additional figures.

## Data Availability

Data generated and analyzed during this study is available from the corresponding author upon reasonable request.
