# Supplementary material for: Polymer-mediated surface engineering enables directional growth of RuO2 nanorods on electrospun V2O5 nanofibers
Source: Beilstein J Nanotechnol. 2026 Nov 4;17:1566–73. doi: 10.3762/bjnano.17.106 (PMC13746496; doi:10.3762/bjnano.17.106)
Supplement: Supplementary file 1 [file Beilstein_J_Nanotechnol-17-1566-s001.pdf]

BEILSTEIN JOURNAL OF NANOTECHNOLOGY

Supporting Information

for

Polymer-mediated surface engineering enables directional growth of RuO_2 nanorods on electrospun V_2O_5 nanofibers

Jiwoo Song, Namhee Kim, Minjae Kim, Myung Hwa Kim and Dasol Jin

Beilstein J. Nanotechnol. **2026,** *17,* 1566–1573. doi:10.3762/bjnano.17.106

Additional figures

License and Terms: This is a supporting information file under the terms of the Creative Commons Attribution License (https://creativecommons.org/licenses/by/4.0). Please note that the reuse, redistribution and reproduction in particular requires that the author(s) and source are credited and that individual graphics may be subject to special legal provisions.
The license is subject to the *Beilstein Journal of Nanotechnology* terms and conditions: (https://www.beilstein-journals.org/bjnano/terms)

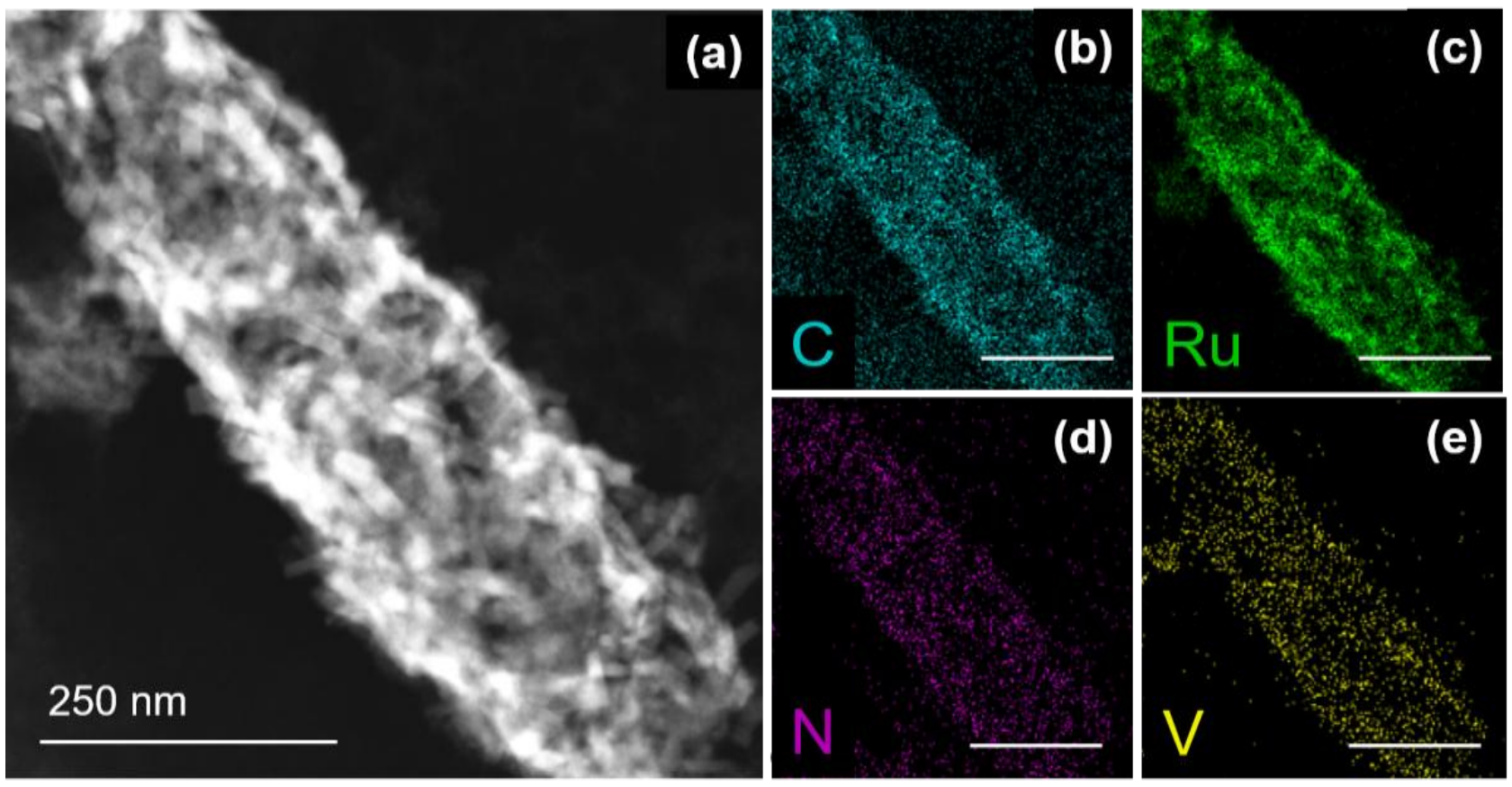

Figure S1: (a) HR-TEM image of RuO_2–VF(0) and the corresponding EDS elemental mapping images of (b) C, (c) Ru, (d) N and (e) V.

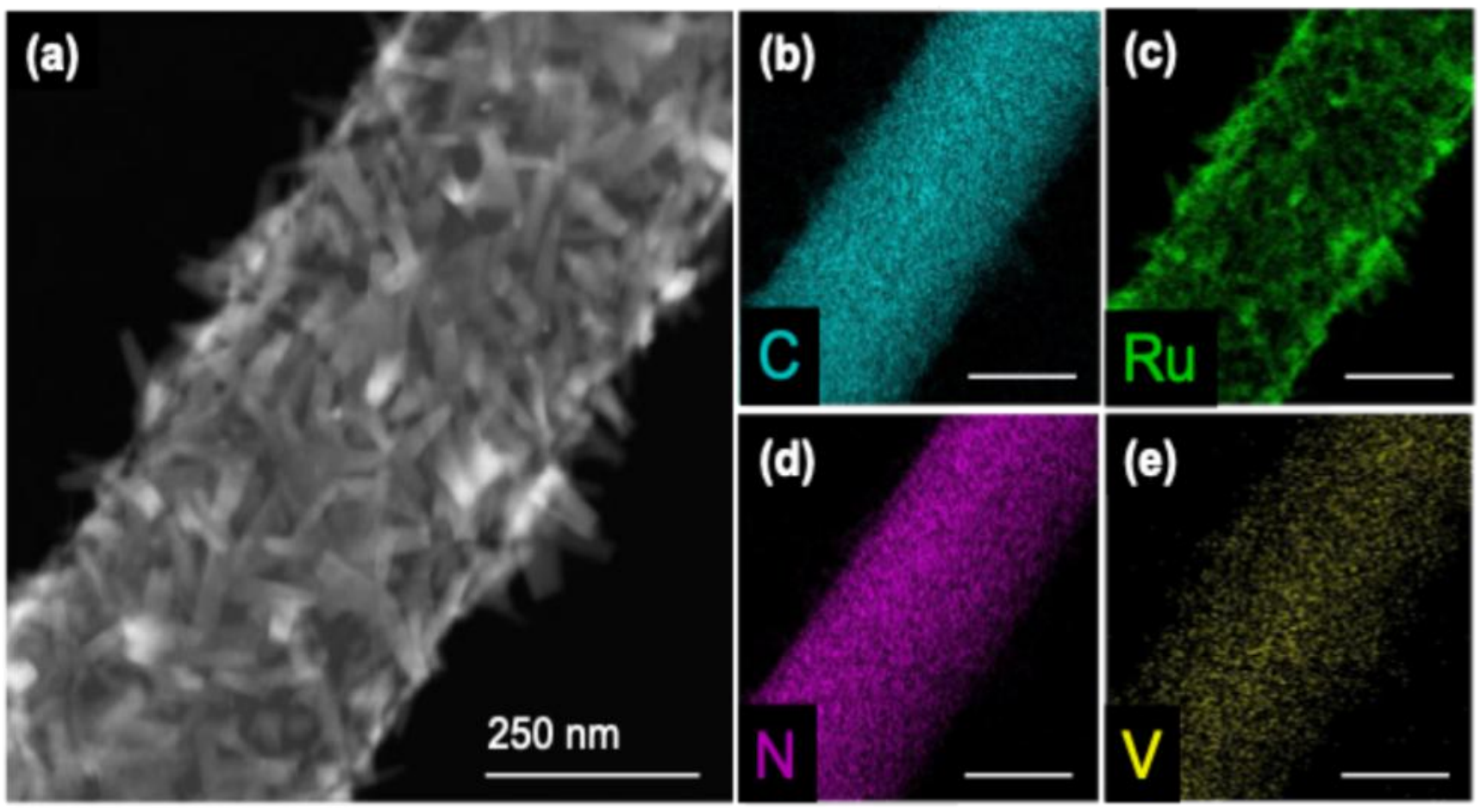

Figure S2: (a) HR-TEM image of RuO_2–VF(50) and the corresponding EDS elemental mapping images of (b) C, (c) Ru, (d) N and (e) V.

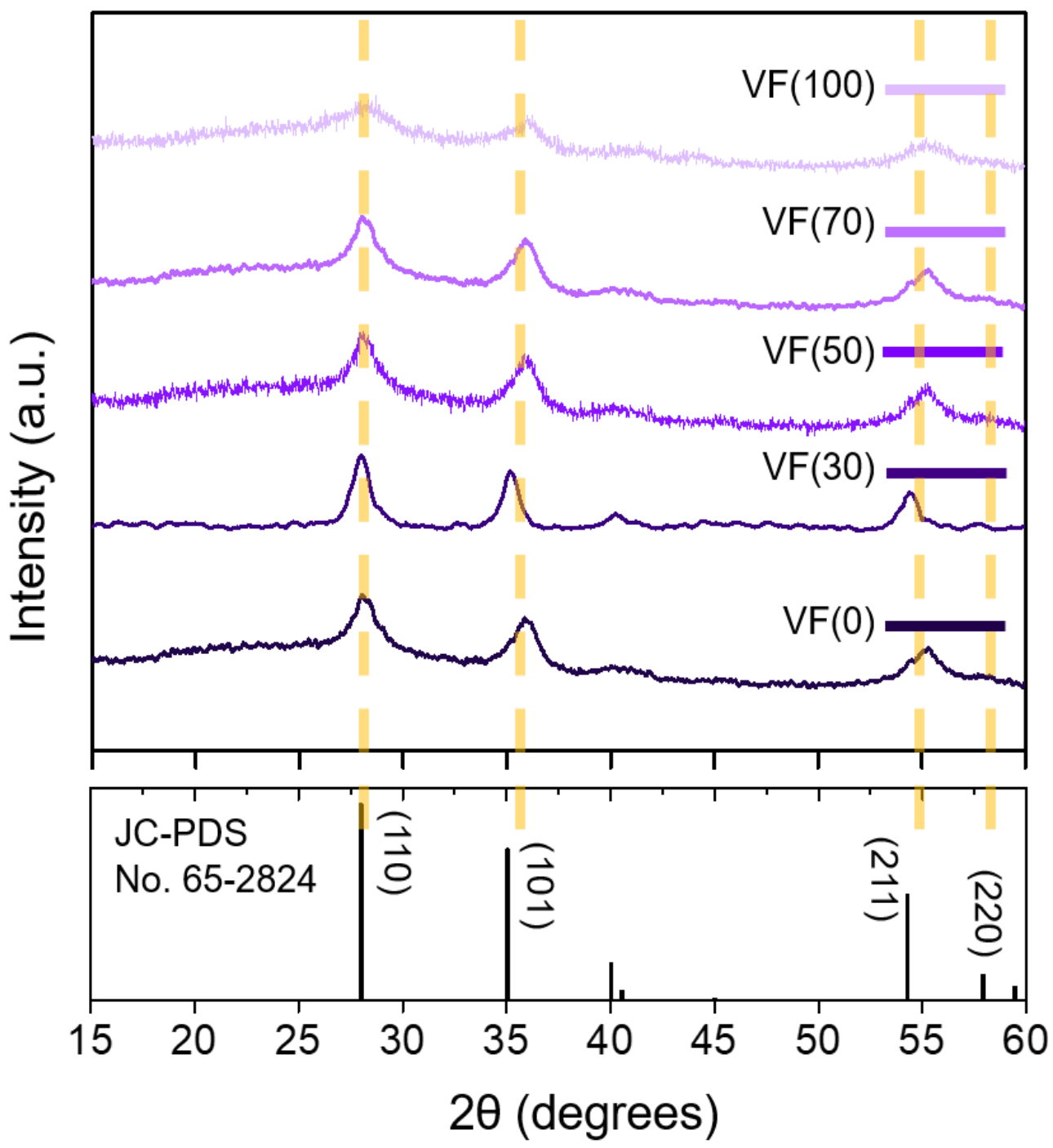

Figure S3: XRD pattern of RuO_2 with diffraction peaks indexed according to JCPDS No. 65-2824.
